# Species-specific regulation of angiogenesis by glucocorticoids reveals contrasting effects on inflammatory and angiogenic pathways

**DOI:** 10.1371/journal.pone.0192746

**Published:** 2018-02-15

**Authors:** Ruth Morgan, John Keen, Daniel Halligan, Alan O’Callaghan, Ruth Andrew, Dawn Livingstone, Amber Abernethie, Giorgia Maltese, Brian Walker, Patrick Hadoke

**Affiliations:** 1 University/ BHF Centre for Cardiovascular Science, The Queen’s Medical Research Institute, University of Edinburgh, Edinburgh, United Kingdom; 2 Royal (Dick) School of Veterinary Studies, University of Edinburgh, Edinburgh, United Kingdom; 3 Fios Genomics Ltd, Nine Edinburgh Bioquarter, Edinburgh, United Kingdom; Ottawa Hospital Research Institute, CANADA

## Abstract

Glucocorticoids are potent inhibitors of angiogenesis in the rodent *in vivo* and *in vitro* but the mechanism by which this occurs has not been determined. Administration of glucocorticoids is used to treat a number of conditions in horses but the angiogenic response of equine vessels to glucocorticoids and, therefore, the potential role of glucocorticoids in pathogenesis and treatment of equine disease, is unknown. This study addressed the hypothesis that glucocorticoids would be angiostatic both in equine and murine blood vessels.The mouse aortic ring model of angiogenesis was adapted to assess the effects of cortisol in equine vessels. Vessel rings were cultured under basal conditions or exposed to: foetal bovine serum (FBS; 3%); cortisol (600 nM), cortisol (600nM) plus FBS (3%), cortisol (600nM) plus either the glucocorticoid receptor antagonist RU486 or the mineralocorticoid receptor antagonist spironolactone. In murine aortae cortisol inhibited and FBS stimulated new vessel growth. In contrast, in equine blood vessels FBS alone had no effect but cortisol alone, or in combination with FBS, dramatically increased new vessel growth compared with controls. This effect was blocked by glucocorticoid receptor antagonism but not by mineralocorticoid antagonism. The transcriptomes of murine and equine angiogenesis demonstrated cortisol-induced down-regulation of inflammatory pathways in both species but up-regulation of pro-angiogenic pathways selectively in the horse. Genes up-regulated in the horse and down-regulated in mice were associated with the extracellular matrix. These data call into question our understanding of glucocorticoids as angiostatic in every species and may be of clinical relevance in the horse.

## Introduction

Angiogenesis, the formation of new blood vessels from existing vasculature, is essential for tissue repair [1]. Aberrant angiogenesis is an important feature of several disease processes including the growth of tumours [2], diabetic retinopathy [3] and rheumatoid arthritis [4]. Glucocorticoids at supra-physiological levels and in the presence of heparin, are potent inhibitors of angiogenesis in the chick embryo and rabbit corneal models [5]. At physiological concentrations, glucocorticoids inhibit angiogenesis in rodent models, both *in vitro* and *in vivo* [6]. When first described, this angiostatic effect presented a potentially significant therapeutic breakthrough in the prevention of tumour metastasis and aberrant angiogenesis [5, 7]. In addition, reduced angiogenesis is described in circumstances of chronic exposure to excess endogenous or exogenous glucocorticoids [8–10]. There has, however, been limited use of glucocorticoids as angiogenesis inhibitors in human medicine [11–13].

Glucocorticoids are frequently administered to veterinary species such as horses, in which prednisolone and dexamethasone are commonly prescribed for allergic dermatological and respiratory conditions. In horses glucocorticoids are also used for the initial treatment of tumours such as lymphoma but with limited success [14]. Glucocorticoid administration, and dysregulation of glucocorticoids in Equine Cushing’s Disease, have been implicated in the development of the vascular condition of the hoof, laminitis [15–17]. In chronic laminitis the blood vessels of the hoof fail to regenerate and there is evidence of a blunted angiogenic response with attenuation of the blood vessels and filling defects [18, 19]. The angiogenic response of equine vessels to glucocorticoids and, therefore, the potential role of glucocorticoids in pathogenesis and treatment of equine disease is unknown.

The angiostatic effect of glucocorticoids is mediated by the glucocorticoid receptor in rodents [6] and in human endothelial cells [20] but the target cell and mechanism is unclear. Shikatani *et al*. found that corticosterone-treated rat endothelial cells exhibited reduced migration, through reduced RhoA and MMP-2 mediated proteolysis [21]. Migration of rat vascular smooth muscle cells and their MMP2 activity is also inhibited by dexamethasone but this effect is not observed in human smooth muscle cells [22]. Glucocorticoids prevent tube-like structure formation by human endothelial cells [20]. Logie *et al*. demonstrated that cortisol induces cytoskeletal disruption, interfering with cell-to-cell contact of endothelial cells, but does not inhibit their proliferation or migration [20]. Analysis of a selection of candidate genes in that study showed only induction of anti-angiogenic thrombospondin-1 [20]. There is also evidence that glucocorticoids, by activating macrophages or myofibroblasts, can mediate a paracrine effect on endothelial cells which alters their angiogenic state [23, 24].

There is marked variation in response to glucocorticoids between cells, species and models of angiogenesis [22, 25]. Comparing contrasting effects of glucocorticoids in different models may provide insights into a final common pathway for glucocorticoid-induced angiostasis. In this study we compared the effects of glucocorticoids on vessels of different species (horses and mice) in a whole vessel model; hypothesising that glucocorticoids would be angiostatic in both species. We hypothesised that a next generation sequencing approach would reveal previously unidentified pathways important in glucocorticoid-mediated effects on angiogenesis.

## Materials and methods

### Drugs

Unless otherwise stated, chemicals, reagents and drugs were obtained from Sigma, Dorset, UK.

### Animals

This study was approved by the University of Edinburgh Veterinary Ethical Review Committee. Healthy horses (n = 10) and horses with laminitis (n = 9), destined for euthanasia, were recruited from clinics at the Royal (Dick) School of Veterinary Studies. Females and castrated males were included, reflecting the clinical population in the UK. Blood was obtained after overnight fasting, between 0900h and 1100h, via an intravenous cannula inserted in the jugular vein for the purpose of euthanasia. Horses were humanely euthanased with intravenous quinalbarbitone sodium and cinchocaine hydrochloride (1mL/10kg bodyweight; Somulose, Dechra Veterinary Products, Shrewsbury, UK).

Adrenocorticotrophic hormone (ACTH), cortisol and insulin concentrations were measured by chemiluminescent immunoassays validated for clinical use in the horse (Immulite 2000, Siemens, Camberley, UK). See supplementary data (Tables A and B in S1 File) for clinical and biochemical characteristics of the horses included.

All murine investigations were approved by the institutional ethical committee and performed under the Provisions of the Animals Scientific Procedures Act (1986) of the UK Home Office, in accordance with EU Directive 2010/63. Five male C57BL6/J mice aged 8 weeks (Charles River Laboratories International Inc., Massachusetts, US) were sacrificed by CO_2_ asphyxiation.

### Tissue Preparation

Thoracic aortae were removed from the mice. Subcutaneous facial skin arteries (50–100μm in diameter) and laminar arteries and veins (100–500μm in diameter) were harvested from horses [26]. Following dissection the vessels were kept in physiological saline solution at 4°C while any adherent adipose and connective tissue was removed and 1 mm rings were prepared. Embedding was achieved within 2 hours of collection.

To quantify angiogenesis vessel rings were embedded in Matrigel (250μl, BD Biosciences, Oxford, UK) and incubated at 37°C (5% CO_2_) in serum free Dulbecco’s Modified Eagle Medium (DMEM, Lonza Group Ltd., Basel, Switzerland) with heparin and ascorbic acid in the presence or absence of serum (foetal bovine serum 3%), cortisol (600nM), serum with cortisol, cortisol with the glucocorticoid receptor antagonist RU38486 (10^−6^ M) or cortisol with the mineralocorticoid receptor antagonist spironolactone (10^−6^ M). Drugs were dissolved in ethanol and diluted in DMEM: final ethanol concentration 1–3% vol/vol. The media were changed every 48 hours. Experiments were performed in triplicate. New vessels were counted, using inverted light microscopy, on days 3, 5 and 7. To confirm the nature of the new vessels, the embedded vessels were subjected to immunohistochemical analysis on day 5. The Matrigel-embedded vessels were fixed in zinc formalin and stained for CD31 (AB28364, Abcam plc, Cambridge, UK).

### Statistical analysis

Two way ANOVAs with Bonferroni post-hoc tests were used to determine the effect of embedding matrix, vessel type (laminar artery *vs*. vein) and anatomical site (facial *vs*. laminar) on the response to treatment of vessels. The mean outgrowth numbers per treatment group at each time point were compared using a one-way ANOVA and Dunnett’s post-hoc test comparing all treatment to DMEM. All analyses were carried out in Graph Pad Prism 4 or SPSS Statistics 19. Data are expressed as mean +/- SEM (n = number of horses).

### Next generation-sequencing of vessel rings from mice and horses incubated with FBS or FBS and cortisol

Vessels isolated from healthy horses (n = 3) and C57BL6/J mice (male, 8 weeks old, n = 3), embedded in collagen, were mechanically disrupted in QIAzol (Qiagen Inc, Valencia, CA, USA) after 5 days culture in medium containing FBS or FBS plus cortisol. Total RNA was extracted using the RNAeasy Mini Kit (Qiagen Inc,). RNA quantity and quality were evaluated using the Agilent 2100 Bioanlyser (Agilent Technologies, California, USA). RNA was deemed of sufficient quality if the RNA integrity number was >7.0.

RNA from each of the samples was profiled on an Illumina HiSeq sequencer. Adapters were trimmed using CutAdapt (http://journal.embnet.org/index.php/embnetjournal/article/view/200), after which the reads were aligned to their respective genomes using TopHat2 (http://genomebiology.biomedcentral.com/articles/10.1186/gb-2013-14-4-r36): mouse genome version GRCm38 for murine RNA and horse genome version EquCab2 for equine RNA. Read counts were generated with HTSeq-Count (http://www.ncbi.nlm.nih.gov/pubmed/25260700) and transformed to log_2_ counts per million reads using voom (http://genomebiology.biomedcentral.com/articles/10.1186/gb-2014-15-2-r29).

### Statistical analysis

Following transformation from raw counts per gene to log_2_ counts per million reads, samples were assessed for quality using the array Quality Metrics R package (http://www.ncbi.nlm.nih.gov/pubmed/19106121) and principal component analysis. No quality issues were identified within the data. The data were then normalised using a TMM (Trimmed Mean of M-component) approach (https://genomebiology.biomedcentral.com/articles/10.1186/gb-2010-11-3-r25).

The factors in the experimental design were assessed to determine whether any were confounded with each other by calculating all pairwise associations per sample. Furthermore, the per-sample associations between each factor and the principal components of the (normalised) expression data were determined. A moderate association (p < 0.01 in a linear model) was observed between the individual from which each RNA sample was taken and principal component 1, as expected when using non-clonal animals. However, the presence of paired FBS and cortisol-treated samples limits the impact that this association may have on downstream analysis.

Statistical analysis was subsequently performed using empirical Bayes from the limma R package (http://m.nar.oxfordjournals.org/content/early/2015/01/20/nar.gkv007.abstract). TMM normalised data in the form of log_2_ counts per million reads provide the input for statistical hypothesis testing. For murine and equine samples, separate statistical comparisons were undertaken using linear modelling. For each species, a single statistical contrast was performed of samples cultured in FBS and cortisol relative to samples cultured in FBS only. Subsequently, empirical Bayesian moderation was applied using limma. For each comparison, the null hypothesis was that there was no difference between the groups being compared. Due to the small sample size and the impact of individual variation on the first principal component, as noted previously, a relaxed statistical significance threshold comprising a fold change ≥ 2 and p < 0.01 was employed, without adjustment for multiple testing. Due to the lack of adjustment for multiple testing, it is possible that many of the differentially-expressed genes identified are false positives. Further analysis was therefore performed to identify enrichment of whole pathways on exposure to cortisol.

Following identification of putatively differentially-expressed genes, the Kyoto Encyclopaedia of Genes and Genomes (KEGG) pathways [27] and Gene Ontology (GO) terms [28, 29] were assessed for pathway enrichment.

Significant genes with raw p < 0.01 and fold change ≥2 from each comparison were analysed for enrichment of GO terms across all three GO ontologies using a hypergeometric test. No correction was applied for multiple testing, nor for the structure of the GO graph. Enrichment (p < 0.05) was assessed for up- and down-regulated genes separately.

### Validation of next generation sequencing

Gene expression changes identified by next generation sequencing analysis were validated by real time quantitative PCR of RNA extracted. Validation genes chosen included those identified as differentially expressed in mice compared to horses (*Scube2*, *Comp*, *Pla1*, *Rasl12*) and genes known to be regulated by glucocorticoids (*Per1*, *Fkbp51*, *Col4a1*, *Cxc*,*5*). Total RNA was extracted from embedded murine aortae incubated with vehicle or cortisol (1μM, n = 5) (Qiagen Inc, Valencia, CA, USA). Four aortic rings were combined and mechanically disrupted in QIAzol (Qiagen). Total RNA was extracted using an RNeasy Mini Kit according to the manufacturer’s instructions. cDNA was synthesised from 75ng RNA using a high Capacity cDNA Reverse Transcription Kit with RNAse Inhibitor (Applied Biosystems, Lithuania) according to the manufacturer’s instructions.

Quantitative real-time polymerase chain reaction was performed using a Light-cycler 480 (Roche Applied Science, Indianapolis, IN, USA). Primers were designed using sequences from the National Centre of Biotechnological Information and the Roche Universal Probe Library (see Table C in S1 File for details of primers for genes of interest and housekeeping genes). Samples were analysed in triplicate and amplification curves plotted (y axis fluorescence, x axis cycle number). Triplicates were deemed acceptable if the standard deviation of the crossing point was < 0.5 cycles. A standard curve (y axis crossing point, x axis log concentration) for each gene was generated by serial dilution of cDNA pooled from different samples and fitted with a straight line and deemed acceptable if reaction efficiency was between 1.7 and 2.1.

## Results

**Fig 1:**
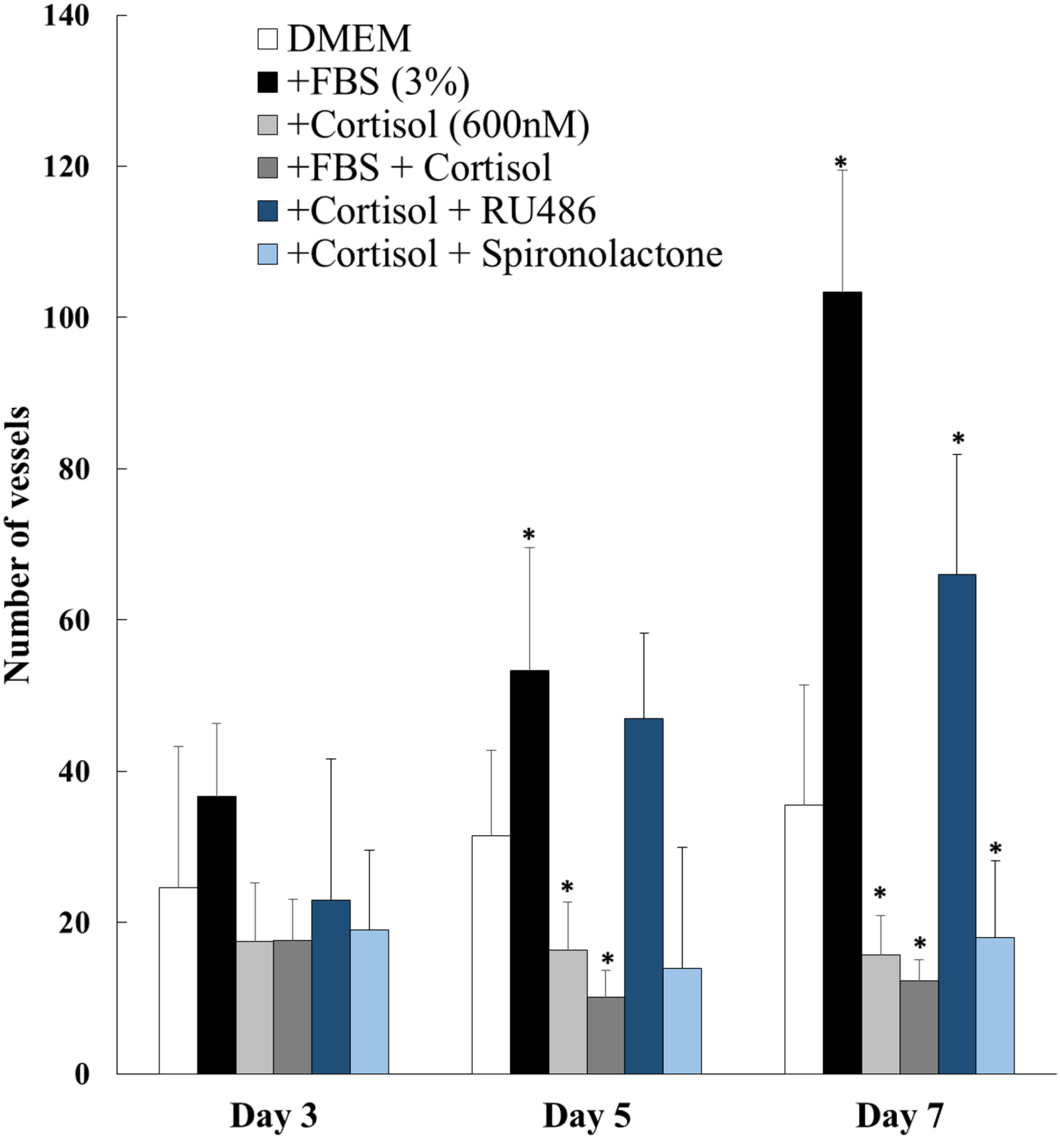
Cortisol inhibits angiogenesis in murine aortae. New vessel outgrowths from murine aortae (C57BL/6J, male, 8 weeks of age, n = 10) in the presence of DMEM, foetal bovine serum (FBS), cortisol, FBS+ cortisol, cortisol + RU486, or cortisol + spironolactone. Data are mean ± SEM and were analysed by one-way ANOVA and Dunnett’s post-hoc test at each time point. * P<0.05in comparison to DMEM.

New vessel growth from murine aortae was stimulated by foetal bovine serum (FBS; Fig 1). Cortisol inhibited both basal and FBS-induced growth of new vessels from murine aortae (Fig 1). This inhibitory effect was abolished by antagonism of glucocorticoid, but not mineralocorticoid, receptor (Fig 1).

**Fig 2:**
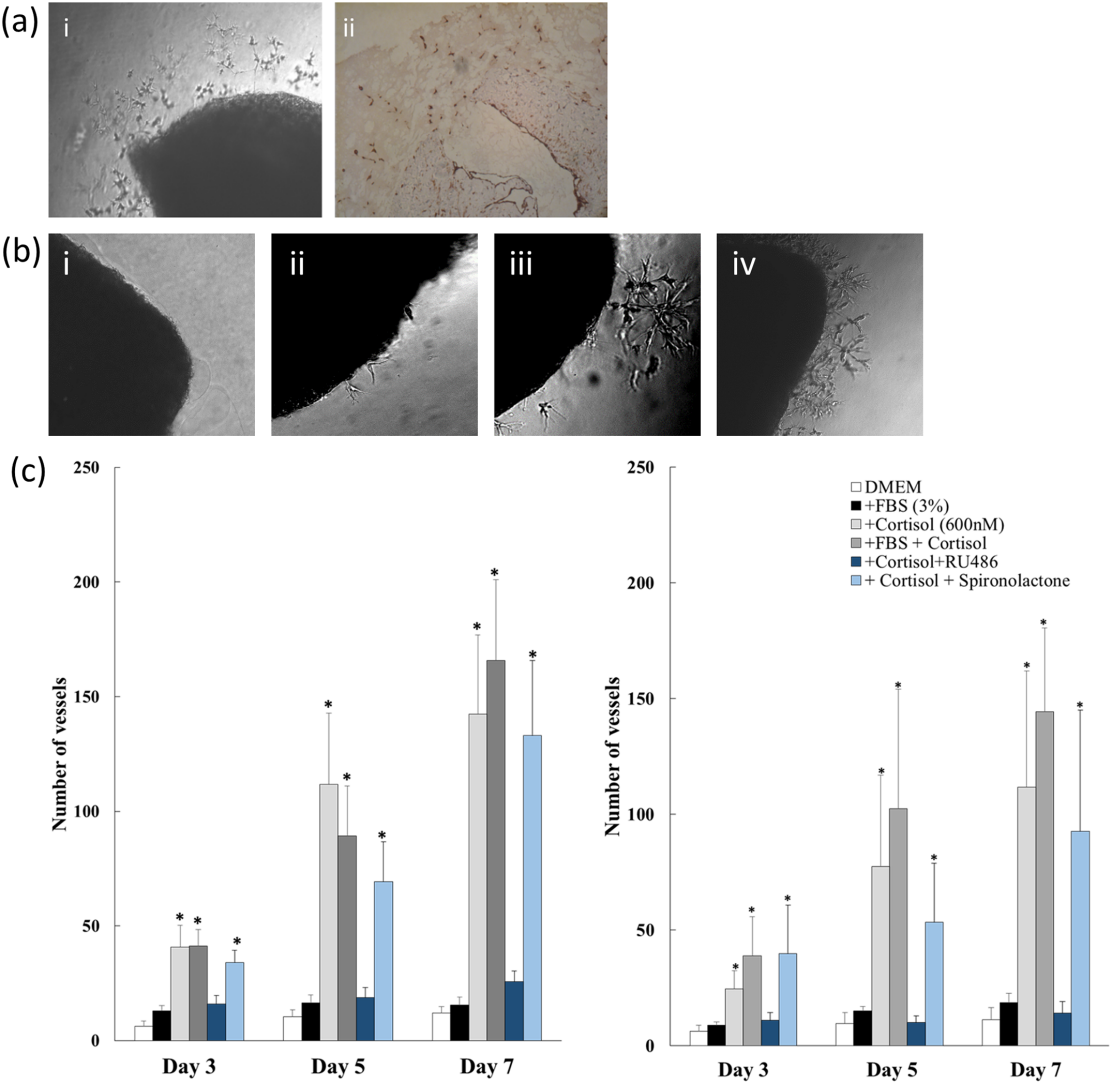
Cortisol stimulates angiogenesis in equine vessels. (a) Light microscopy images of new vessel outgrowths [i], which stained strongly (brown) for CD31 [ii], indicating they are likely to be predominantly endothelial in nature (Scale 0.2mm). (b) Light microscopy images of equine laminar vessel sections after incubation (5 days) with DMEM [i], foetal bovine serum [ii], cortisol [iii], or FBS with cortisol [iv]. These demonstrate the stimulatory effect of cortisol on new vessel growth from equine vessels. (c) New vessel outgrowths from laminar (n = 10) [i] and facial skin vessels (n = 10) [ii] of healthy horses were quantified in the presence of DMEM, foetal bovine serum (FBS), cortisol, FBS+ cortisol, cortisol + RU486, or cortisol + spironolactone. Data are mean ± SEM for (n) horses) and were analysed by one-way ANOVA and Dunnett’s post-hoc test at each time point. * P<0.05 in comparison to DMEM.

Laminar and facial skin vessels from healthy horses produced vessel outgrowths (Fig 2ai), that stained strongly for CD31 (Fig 2aii), and were similar whether vessels were embedded in Matrigel or collagen (Tables D and E in S1 File). Data from laminar arteries and veins were combined for analysis as there were no significant differences in their response to any of the treatments (Tables F and G in S1 File).

In contrast to its effects on murine aorta, FBS (3%) did not increase basal outgrowth of new vessels from equine vessels (Fig 2bii & 2ci). Furthermore, exposure to cortisol dramatically increased new vessel formation from equine vessels compared with DMEM alone or with FBS (p < 0.001), in both laminar (Fig 2bi, 2bii and 2ci) and facial skin (Fig 2cii) vessels. This increase was evident after 3 days in culture and was maintained at days 5 and 7. The combination of cortisol with FBS produced a similar increase in new vessel formation to cortisol alone (p < 0.001 compared with DMEM or FBS alone) in laminar and facial skin vessels (Fig 2ci and 2cii). There were no differences in vessel growth in response to treatment between healthy horses and horses with laminitis (S1 Fig).

Exposure to antagonists targeted at GR (RU38486) or MR (spironolactone) had no effect on basal (DMEM alone) new vessel formation from equine vessels (data not shown). However, RU38486 inhibited the cortisol-stimulated formation of new vessels (p = 0.003) whereas spironolactone did not (Fig 2ci and 2cii).

### Transcriptomic analysis

#### The effect of cortisol on the transcriptome of murine vessels

In total, 341 genes were differentially expressed when FBS-stimulated murine vessels were exposed to cortisol (at an unadjusted p-value of ≤ 0.05 and a fold-change ≥ 2).

**Fig 3:**
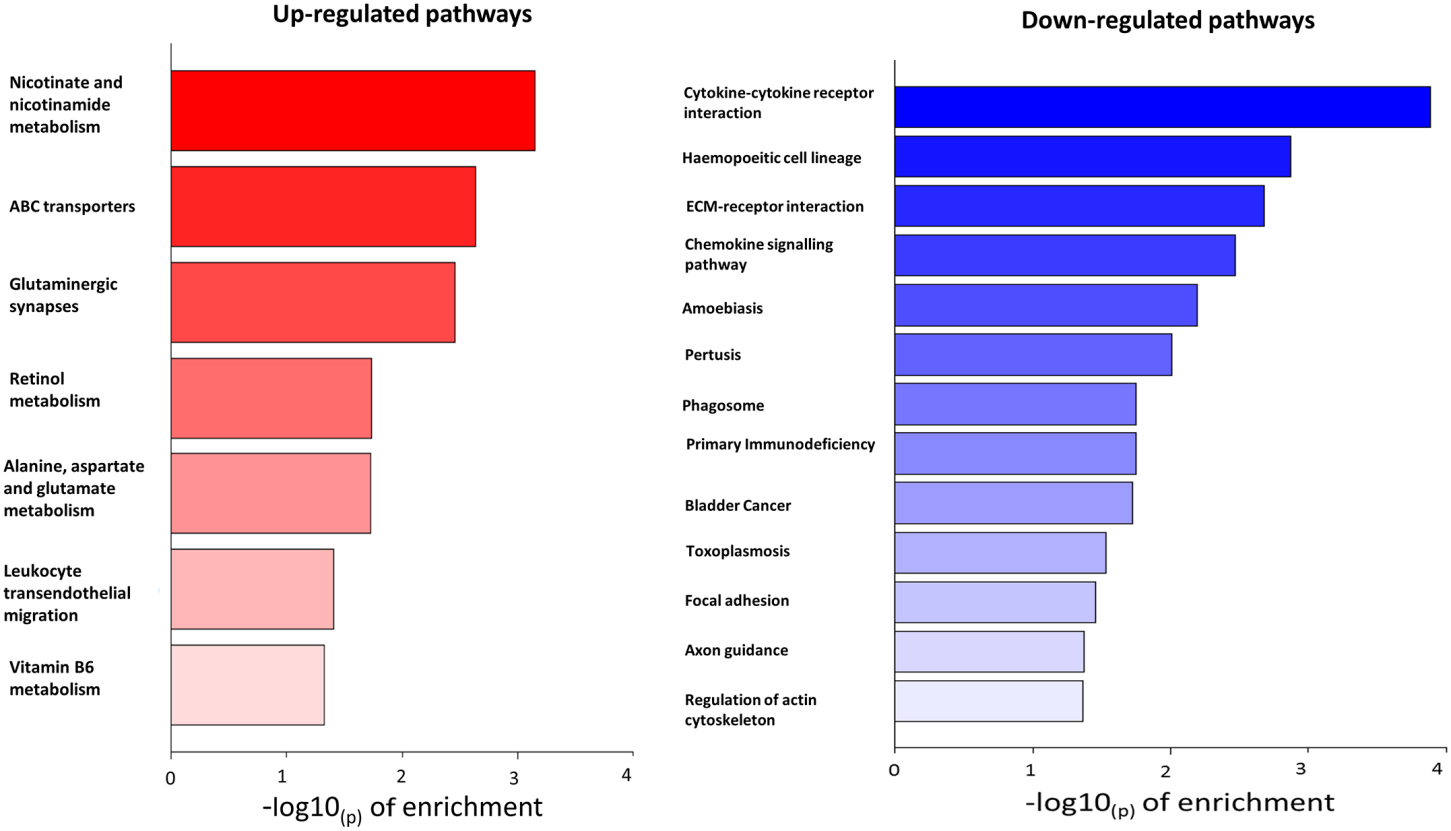
KEGG enrichment analysis of pathways up- or down-regulated in murine aortae in response to cortisol. Twenty KEGG pathways were identified as significantly enriched (at an unadjusted p-value of 0.05). Of these, 7 were enriched for up-regulated genes (red), and 13 for down-regulated genes (blue). Of the pathways down-regulated by cortisol 9/13 were associated with inflammatory or immune responses and 4 were associated with extracellular matrix or cytoskeletal function.

Twenty KEGG pathways were identified as significantly enriched (at an unadjusted p-value of ≤ 0.05). Of these, 7 were enriched for up-regulated genes, and 13 for down-regulated genes (Fig 3). Of the 13 pathways down-regulated in cortisol-treated tissue, 9 were associated with inflammatory or immune responses and 4 were associated with extracellular matrix or cytoskeletal function. GO terms were significantly enriched for 483 up-regulated genes, and for 485 down-regulated genes (using unadjusted p-values of ≤ 0.05; Table 1 shows the top 8 up- or down-regulated genes). Pathways specifically related to angiogenesis (such as VEGF signalling) were not altered by exposure to cortisol. Many of the GO terms enriched among the up-regulated genes were related to transmembrane transport and homeostasis, as well as peptidase activity. Within the down-regulated GO terms there was a predominance of immune response/inflammation pathways.

**Table 1:** GO term enrichment analysis comparing murine samples incubated with FBS (control) with those incubated with cortisol. The top 8 up-regulated and down-regulated pathways are shown.

Pathways up-regulated in the presence of cortisol (P<0.05)	Pathways down-regulated in the presence of cortisol (P<0.05)
Metal ion transport	Extracellular space
Dipeptidase activity	Extracellular region
Cation transport	Killing of cells of other organism
Ion transport	Disruption of cells of other organism
Sodium ion transport	Extracellular region part
Drug metabolic process	Modification of morphology or physiology of other organism
Dipeptidyl-peptidase activity	Cell adhesion
Transition metal ion homeostasis	Regulation of killing of cells of other organism

### The effect of cortisol on the transcriptome of equine vessels

**Fig 4:**
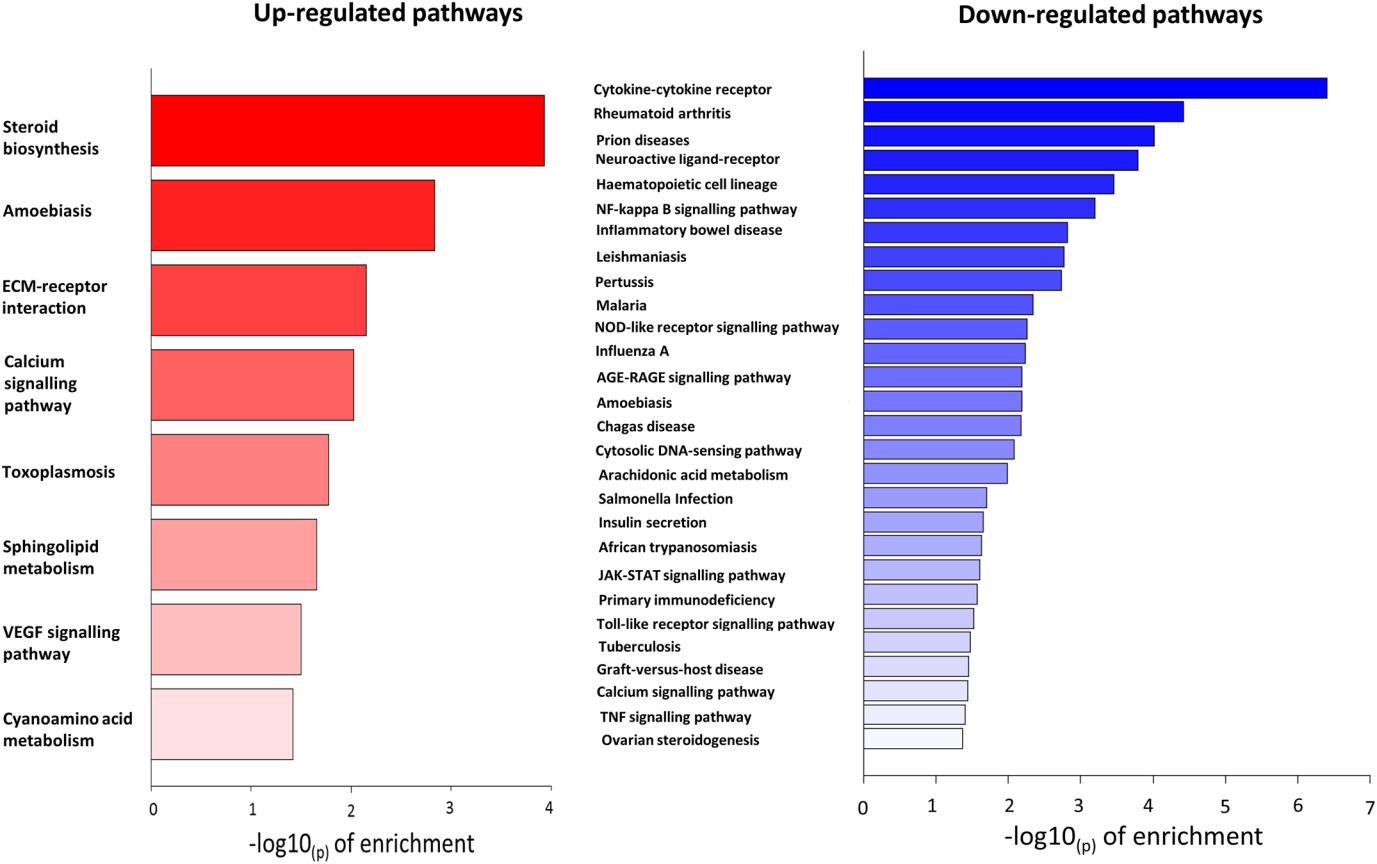
KEGG enrichment analysis of pathways up- or down-regulated in equine laminar vessels in response to cortisol. Thirty six KEGG pathways were identified as significantly enriched. Eight were up-regulated (red) and 28 were down-regulated (blue).

Cortisol exposure induced differential expression of a total of 246 genes in equine vessels. Thirty six KEGG pathways were identified as significantly enriched. Eight were up-regulated and 28 were down-regulated (Fig 4). Up-regulated pathways were diverse but included calcium signalling and sphingolipid metabolism as well as VEGF signalling. Within the up-regulated pathways, the most commonly overlapping differentially-expressed genes included LAMA2, LAMC3 and SPHK1. Of those pathways down-regulated by cortisol 18/28 were associated with inflammatory or immune responses, a similar response to that observed in murine vessels.

One hundred and fifty-five GO terms were identified as being significantly up-regulated and 331 down-regulated by cortisol. Within the top 8 up-regulated pathways (Table 2) were ‘endothelial cell migration’ and ‘vena cava morphogenesis’ which include specific angiogenic signalling. Interestingly, ‘blood vessel development’ was among the top 8 down-regulated pathways in the GO analysis, suggesting that not all angiogenic pathways were up-regulated by cortisol in equine vessels.

**Table 2:** GO term enrichment analysis comparing equine samples incubated with FBS (control) with those incubated with cortisol. The top 8 up-regulated and down-regulated pathways are shown.

Pathways up-regulated in the presence of cortisol (P<0.05)	Pathways down-regulated in the presence of cortisol (P<0.05)
Negative regulation of protein kinase activity	Extracellular region
Nucleosome	Cytokine activity
Regulation of I-kappaB kinase/NF-kappaB signalling	Regulation of heart rate by cardiac conduction
Positive regulation of blood vessel endothelial cell migration	Inflammatory response
Integral component of plasma membrane	Interleukin-1 receptor binding
Superior vena cava morphogenesis	N-formyl peptide receptor activity
Subthalamic nucleus development	Embryonic digestive tract development
Chromosome	Blood vessel development

### A comparison of the murine and equine responses to cortisol

There was little congruence between the pathway analysis of murine and equine angiogenesis models in their response to cortisol.

Seven KEGG pathways overlapped between the two species: "Amoebiasis", "Toxoplasmosis", "ECM-receptor interaction", "Primary immunodeficiency", "Pertussis", "Hematopoietic cell lineage", and "Cytokine-cytokine receptor interaction", all of which were downregulated by cortisol in both species. All of these pathways have inflammatory or immune responses as their key elements.

Fifty-five GO terms overlapped between the species in response to cortisol when not considering direction of change; no more than would be expected by chance. Furthermore, the distribution of these terms between being disturbed in the same direction (both up or both down) and in different directions (one up and one down) was roughly equal. This implies there was no clear pattern of differential regulation of GO terms revealed by this analysis.

Analysis of individual genes differentially expressed in horses and mice in response to cortisol identified 18 genes that were up-regulated by cortisol in the horse and down-regulated in the mouse (Table 3). GO analysis of these genes showed enrichment of the ‘extracelluar matrix’ GO term. 10 genes were down-regulated in the horse and up-regulated in the mouse (Table 4). GO analysis did not show any pathway enrichment for these genes.

**Table 3:** Genes identified by next-generation sequencing that were up-regulated in the horse by cortisol and down-regulated in the mouse.

Gene Symbol	Gene Name	Horse log fold change	Horse adjusted p value	Mouse log Fold change	Mouse adjusted p value
*Comp*	Cartilage oligomeric matrix protein	3.5	0.009	-2.4	1.1E-05
*Creb3l1*	cAMP responsive element binding protein 3-like 1	1.7	4.8E-08	-0.7	0.01
*Cxcl14*	Chemokine (C-X-C motif) ligand 14	1.2	0.02	-2.8	2.5E-10
*Fam65c*	Family with sequence similarity 65, member C	3.3	3.1E-07	-1.4	0.0005
*Fbln5*	Fibulin 5	1.9	0.0018	-1.0	0.0005
*Gpc4*	Glypican 4	2.2	3.3E-07	-0.6	0.003
*Gprin3*	GPRIN family member 3	3.3	0.0001	-2.4	9.2E-05
*Kcnj15*	Potassium inwardly-rectifying channel, subfamily J, member 15	2.3	5.3E-16	-1.4	0.007
*Matn4*	Matrilin 4	2.6	0.003	-1.3	0.0003
*Mest*	Mesoderm specific transcript	1.0	0.04	-2.1	1.2E-07
*Ptgfr*	Prostaglandin F receptor	1.4	0.0004	-1.2	5.7E-05
*Ptx3*	Pentraxin related gene	4.8	9.6E-08	-1.0	5.5E-07
*Scube2*	Signal peptide, CUB domain, EGF-like 2	0.9	0.006	-3.3	7.1E-06
*Sfrp2*	Secreted frizzled-related protein 2	2.9	1.9E-08	-1.1	0.0004

**Table 4:** Genes identified by next-generation sequencing that were down-regulated in the horse by cortisol and up-regulated in the mouse.

Gene Symbol	Gene Name	Horse log fold change	Horse adjusted p value	Mouse log fold change	Mouse adjusted p value
*Agtrap*	Angiotensin II, type I receptor-associated protein	-1.11	0.007	0.52	0.03
*Birc3*	Baculoviral IAP repeat-containing 3	-1.63	0.002	0.63	0.005
*Fibin*	Fin bud initiation factor homolog (zebrafish)	-1.74	0.01	0.67	0.006
*Gabre*	Gamma-aminobutyric acid (GABA) A receptor, subunit epsilon	-2.29	0.02	1.06	0.0009
*Gem*	GTP binding protein (gene overexpressed in skeletal muscle)	-0.98	0.04	1.11	2.0E-09
*Mnda*	Myeloid cell nuclear differentiation antigen	-1.55	0.0003	0.63	0.02
*Pla1a*	Phospholipase A1 member A	-3.22	0.0001	3.11	4.8E-19
*Rasl12*	RAS-like, family 12	-1.53	0.008	3.72	0.02
*Slco2b1*	Solute carrier organic anion transporter family, member 2b1	-1.64	0.00002	1.30	3.6E-16
*Wdfy1*	WD repeat and FYVE domain containing 1	-1.11	0.007	0.52	0.04

The next generation sequencing analysis was validated by quantification of three genes up-regulated by cortisol (Collagen, type XIV, alpha 1 (*Col4a*), Period 1 (*Per1*) and FK506 binding protein 5 (*Fkbp5*)) and two genes that were down-regulated by cortisol (Matrix metalloprotease 9 (*Mmp9*)) and chemokine (C-X-C motif) ligand 5 (*Cxcl5*)) in the murine model. We demonstrated changes in expression of these genes consistent with the data from next generation sequencing analysis (S2 Fig).

## Discussion

In this study, we demonstrated that cortisol increased angiogenesis in equine vessels in contrast to its angiostatic effects in murine vessels. This unexpected cortisol-dependent induction of angiogenesis was mediated by glucocorticoid receptor (GR).

Our findings in mouse aorta are consistent with previous studies showing that glucocorticoids inhibit angiogenesis in rodent models, via a GR-mediated mechanism, at supra-physiological and physiological concentrations [5–7, 30–32]. Previous literature suggests that, in rodents, glucocorticoids act on multiple steps in the angiogenesis process. Dexamethasone disrupts the cytoskeletal structure and tight-junctions of rat brain endothelial cells [33]. Corticosterone reduces rodent endothelial cell migration and alters proteolysis of the extracellular matrix [21]. *RhoA* and *Mmp2* have been identified as potential candidate genes in the proteolytic process [21]. Indications of species differences have been reported; for example, dexamethasone inhibits migration of rodent vascular smooth muscle cells (VSMC) but not of human VSMCs or endothelial cells [20, 22]. Bovine aortic smooth muscle cells have reduced proliferation when exposed to dexamethasone [34]. There are several potential explanations for the species-specific differences identified in this study. Species-specific differences in GR, particularly variation within the promotor region influencing the gene transactivation potential [35, 36], can result in different responses to ligand binding and are the most likely explanation of this observation. Tissue origin is known to influence the response to glucocorticoids; for example, retinal endothelial cells are more resistant to the toxic effects of glucocorticoids than those of dermal origin [37]. Differences in the composition of the vessel (for example the amount of smooth muscle and the endothelial phenotype) may contribute to the differing responses to cortisol. However, veins of the hoof also showed a cortisol-mediated up-regulation of new vessel growth, suggesting that the observation in the horse is not an idiosyncrasy of a particular equine vessel. The effect of glucocorticoid concentration on response is often non-linear. Data from human ovarian cells lines indicate that cortisol may have an inhibitory effect on VEGF secretion at low concentrations (1nM) but a stimulatory effect at high concentrations (1000nM) [38]. In this assay a concentration of 600 nM was used which, though within the physiological range for humans, is approximately 3–5 times that of equine plasma concentrations. The observed effect may, therefore reflect a “high-dose response” to glucocorticoids rather than a physiological phenomenon.

In chronic laminitis there is marked attenuation of the blood supply and endothelial dysfunction suggesting an inadequate angiogenic response [18, 39]. The current study, however, showed no differences in the angiogenic response to FBS or cortisol between healthy horses and those with laminitis. As previously discussed, FBS did not prove a potent stimulator of angiogenesis in equine vessels and may not, therefore, be a robust measure of angiogenic potential in these vessels. However, in the presence of a stimulator of angiogenesis, in this case cortisol, the number of vessel outgrowths did not differ between the groups. Extrapolation of these findings to the *in vivo* environment should, however, be treated with caution. Vessels within the diseased hoof may be subject to very different environmental conditions, most importantly hypoxia and altered shear stresses, which are not replicated in this model of angiogenesis [40].

It is important to note that whilst this study compared the response of horses to that of mice it did not take into account factors such as age which may affect the angiogenic response in either species. The mice used were young (8 weeks) whilst the horses were old in relation to species life span (average age 19). Aging can have profound effects on both angiogenesis and response to glucocorticoids which may have confounded our results. Aging in humans and rodent models results in a reduction in angiogenesis [41, 42] associated with reduced angiogenic factors such as HIF-1α and VEGF [43, 44] as well as endothelial dysfunction [45]. Such alterations may go some way to explain the poor angiogenic response of equine vessels to the normally stimulatory FBS, further unknown alterations in signalling pathways in aging horses may also contribute to the unusual response to cortisol. Aging is associated in rodents with a reduction in glucocorticoid receptor density and activation; we do not know the effects of aging on GR in the horse.

Given that we saw an opposite effect of cortisol in mice and horses, we looked specifically at genes that were changed in opposite directions between the species. Out of the 14 genes up-regulated in the horse and down-regulated in the mouse, 11 have pro-angiogenic properties. For example, glypican 4 (*Gpc4*) is a cell surface heparan sulfate proteoglycan essential in angiogenesis since this group of proteins bind almost all angiogenic factors to receptors or to inhibitors [46]. Cartilage oligomeric matrix protein (*Comp*), also known as thrombospondin 5, mediates adhesion and migration but not proliferation of vascular smooth muscle cells [47]. cAMP responsive element binding protein 3-like 1 (*Creb3l1*, also known as *Oasis*) is a transcription factor which promotes angiogenesis when complexed with hypoxia-inducible factors [48]. *Cxcl14* is released from mesenchymal stem cells and stimulates cell migration and proliferation and angiogenesis directly [49]. Endothelial *Scube2* potentiates VEGF mediated adult angiogenesis and is suggested to be a novel co-receptor for VEGFR2 [50]. Secreted frizzled-related protein 2 stimulates angiogenesis through the calcineurin/NFAT pathway [51]. Petraxin 3 is highly expressed in endothelial cells and up-regulated in tumour endothelial cells where it plays an important role in promoting proliferation [52]. Prostaglandin F receptor (*Ptgfr*) and Mesoderm specific transcript (*Mest*) are expressed in the placenta and endometrial blood vessels and are thought to play a role in angiogenesis and tissue remodelling during placentation [53] [54]. The matrilins are extracellular matrix proteins belonging to the von Willebrand factor A superfamily [55], which interact with the ADAMT system to induce angiogenesis. Fibulin 5 (*Fbln5*) promotes adhesion of endothelial cells through interaction of integrins and the RGD motif [56]. While these genes all appear to belong to different angiogenesis altering pathways they are predominantly of endothelial origin and encode extracellular proteins (*Comp*, *Fbln5*, *Matn 4*), secreted proteins (*Cxcl14*, *Sfrp2*) or proteins associated with the cell membrane (*Gpc4*, *Ptgfr*, *Scube2*).

These findings suggest that the extracellular matrix is the target for glucocorticoid-induced changes both in horses and in mice, and that classical angiogenesis pathways may not be so important in mediating this effect. GO and KEGG analysis of the whole dataset supports our finding that the extracellular matrix is a key component in the response to glucocorticoids in this system. GO analysis of the murine response demonstrated extracellular pathways and processes were significantly down-regulated. KEGG analysis showed up-regulation in equine and down-regulation in murine samples of the toxoplasmosis, ECM-receptor interaction and amoebiasis pathways. The toxoplasmosis pathway encompasses JAK-STAT signalling as well as extra-cellular matrix interactions and laminins (LAMA2 and LAMC3). Laminins are principal components of the basement membrane and exert tissue-specific effects, whilst both LAMA2 and LAMC3 have angiogenic properties [57]. The ECM-receptor interaction pathway encompasses the integrins (VLA proteins) and their interactions with cell-cell adhesion proteins such as collagen, laminins and fibronectin. The amoebiasis pathway is an inflammatory pathway with a significant extracellular component comprising laminins and collagen proteins. The extracellular matrix (ECM) is a key component of angiogenesis and it is known that the *balance* of angiogenic factors in the extracellular environment, rather than individual proteins, determines whether angiogenesis will occur or not [58]. Changes to the ECM will facilitate or inhibit migration and proliferation of cells but there is growing evidence that the ECM can also directly affect cell behaviour [59–61]. It is widely accepted that glucocorticoids can have direct effects on the ECM and that these effects are often differential and cell-specific [62, 63]; it is therefore not surprising that they are also species-specific.

The genes down-regulated in the horse and up-regulated in the mouse were more varied in their actions and origins, with fewer having previously been reported as associated with angiogenesis. Several were anti-angiogenic (*Agtrap, Gem, Wdfy1* [64–66]) or anti-apoptotic (*Birc3, Mnda* [67, 68]), as demonstrated by their association with tumour metastasis. The complexity of angiogenesis combined with the complexity of GR signalling means that a candidate gene approach to understanding their interaction is limited. To our knowledge this is the first study to apply next generation sequencing technology to this model of angiogenesis and our results provide candidate genes for further exploration and explain why previous, targeted, studies have failed to fully explain the phenomenon of glucocorticoid effects on angiogenesis.

In contrast to the response of mouse aortae in this and other studies [69], there was little increase in new vessel growth when equine vessels were cultured with, the normally pro-angiogenic, FBS. FBS contains a significant number of, largely unidentified, embryonic growth factors which promote cell growth, though the pathways activated are unknown [70]. FBS was used at a low concentration (3%) which has been shown to induce angiogenesis in mouse aortae, a finding repeated in this study [70]. FBS is used successfully to induce proliferation and support growth in other cell and tissue culture systems using equine tissue; for example in cartilage explant models and for the culture of mesenchymal stem cells [71, 72], suggesting there is not an inherent unresponsiveness of equine tissues.

In conclusion, these data demonstrate an unexpected species-specific (GR-mediated) stimulation of angiogenesis by cortisol in equine vessels. We have demonstrated that the extracellular matrix is the key component in the effects of glucocorticoids on angiogenesis. This finding raises important questions about the regulation of angiogenesis and highlights the gaps in our knowledge of the mechanism by which glucocorticoids alter angiogenesis. They may also have significant clinical implications since glucocorticoids are used regularly in horses (though not primarily for their anti-angiogenic actions) for treatment of conditions such as allergic lung and skin disease. Given the limited data available pertaining to human angiogenesis these data also call into question our understanding of glucocorticoids and angiogenesis in this species.

## Supporting information

S1 Fig Laminitis did not affect the response of equine laminar [a] or facial [b] vessels to cortisol. New vessel outgrowths from laminar vessels [a] and facial skin vessels [b] from healthy horses (n = 10) and those with laminitis (n = 6) incubated with DMEM, Foetal Bovine Serum (FBS), cortisol, FBS + cortisol, cortisol + RU486 or cortisol + spironolactone at day 7. Data are mean ± SEM for (n = number of horses) and were analysed by one-way ANOVA and Bonferroni post-hoc test at each time point. There were no differences between healthy horses and those with laminitis.
(TIF)

S2 Fig Gene expression patterns identified by next generation sequencing were validated by RT-qPCR. Next generation sequencing analysis was validated by quantification of three genes known to be up-regulated by cortisol and found to be up-regulated in our sequencing analysis (Collagen, type XIV, alpha 1 (*Col4a*), Period 1 (*Per1*) and FK506 binding protein 5 (*Fkbp5*)) and two genes that are known to be down-regulated by cortisol and were down-regulated in this our sequencing analysis (Matrix metalloprotease 9 (*Mmp9*)) and chemokine (C-X-C motif) ligand 5 (*Cxcl5*)) in the murine model. In addition we validated genes that were differentially expressed in the mouse compared to the horse in our sequencing analysis (*Scube2* (Signal Peptide, CUB Domain, EGF-Like 2) *Comp* (cartilage oligomeric matrix protein), *Pla1a* (Phospholipase A1 member A) and *Rasl12* (RAS-like, family 12). Data are mean ± SEM for and were analysed by Student’s t-test. * = P<0.05.
(TIF)

S1 File **Table A. Clinical and biochemical data from healthy horses and those with laminitis.** (Vessels were cultured in Matrigel for quantification of angiogenic response). **Table B. Clinical and biochemical data from healthy horses and those with laminitis.** (Vessels were cultured in collagen for next generation sequencing). **Table C. Murine primer sequences for PCRS4. Table D. New vessel outgrowths from laminar vessels of healthy horses and those with laminitis cultured in Matrigel or Type 1 Collagen at day 3. Table E. New vessel outgrowths from laminar vessels of healthy horses and those with laminitis cultured in Matrigel or Type 1 Collagen at day 7. Table F. New vessel outgrowths from laminar arteries and laminar veins of healthy horses and those with laminitis cultured in Matrigel at day 3. Table G. New vessel outgrowths from laminar arteries and laminar veins of healthy horses and those with laminitis cultured in Matrigel at day 7.**
(DOCX)
