# Supplementary figures and images for: Species-specific regulation of angiogenesis by glucocorticoids reveals contrasting effects on inflammatory and angiogenic pathways
Source: PLoS One. 2018 Feb 15;13(2):e0192746. doi: 10.1371/journal.pone.0192746 (PMC5813970; doi:10.1371/journal.pone.0192746)

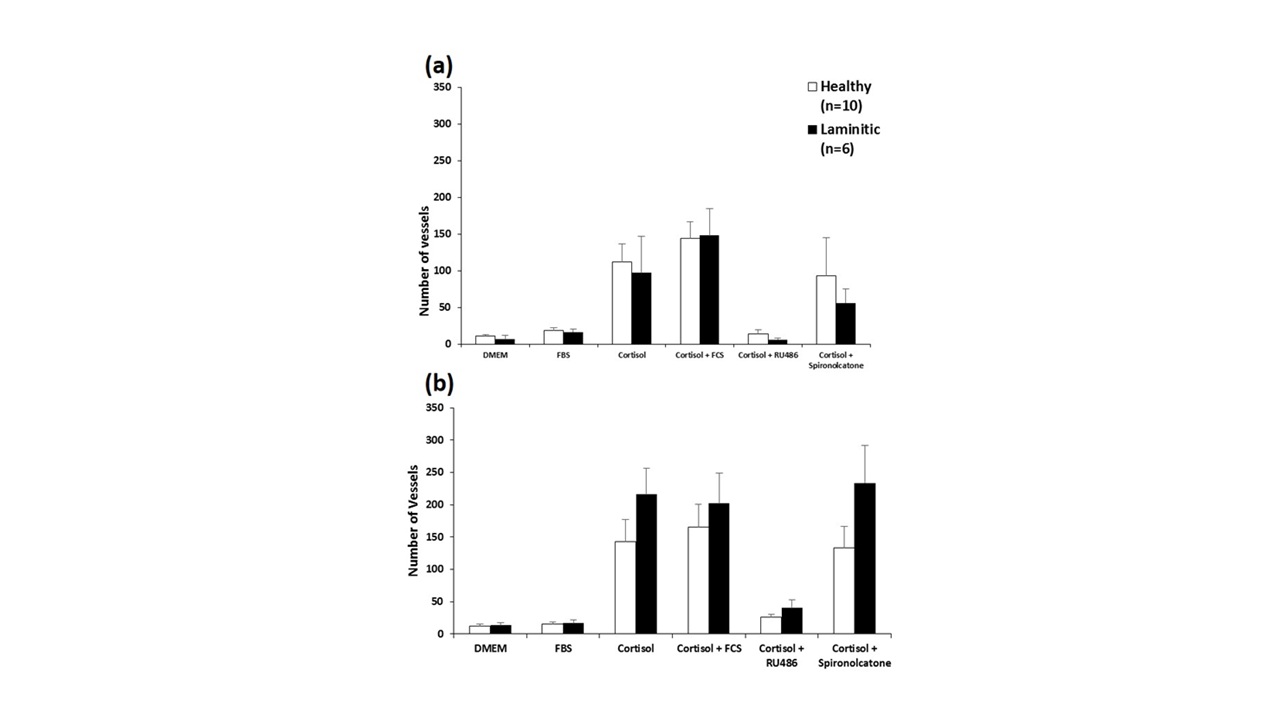

Supplement: S1 Fig — New vessel outgrowths from laminar vessels [a] and facial skin vessels [b] from healthy horses (n = 10) and those with laminitis (n = 6) incubated with DMEM, Foetal Bovine Serum (FBS), cortisol, FBS + cortisol, cortisol + RU486 or cortisol + spironolactone at day 7. Data are mean ± SEM for (n = number of horses) and were analysed by one-way ANOVA and Bonferroni post-hoc test at each time point. There were no differences between healthy horses and those with laminitis. (TIF) [file pone.0192746.s001.tif]

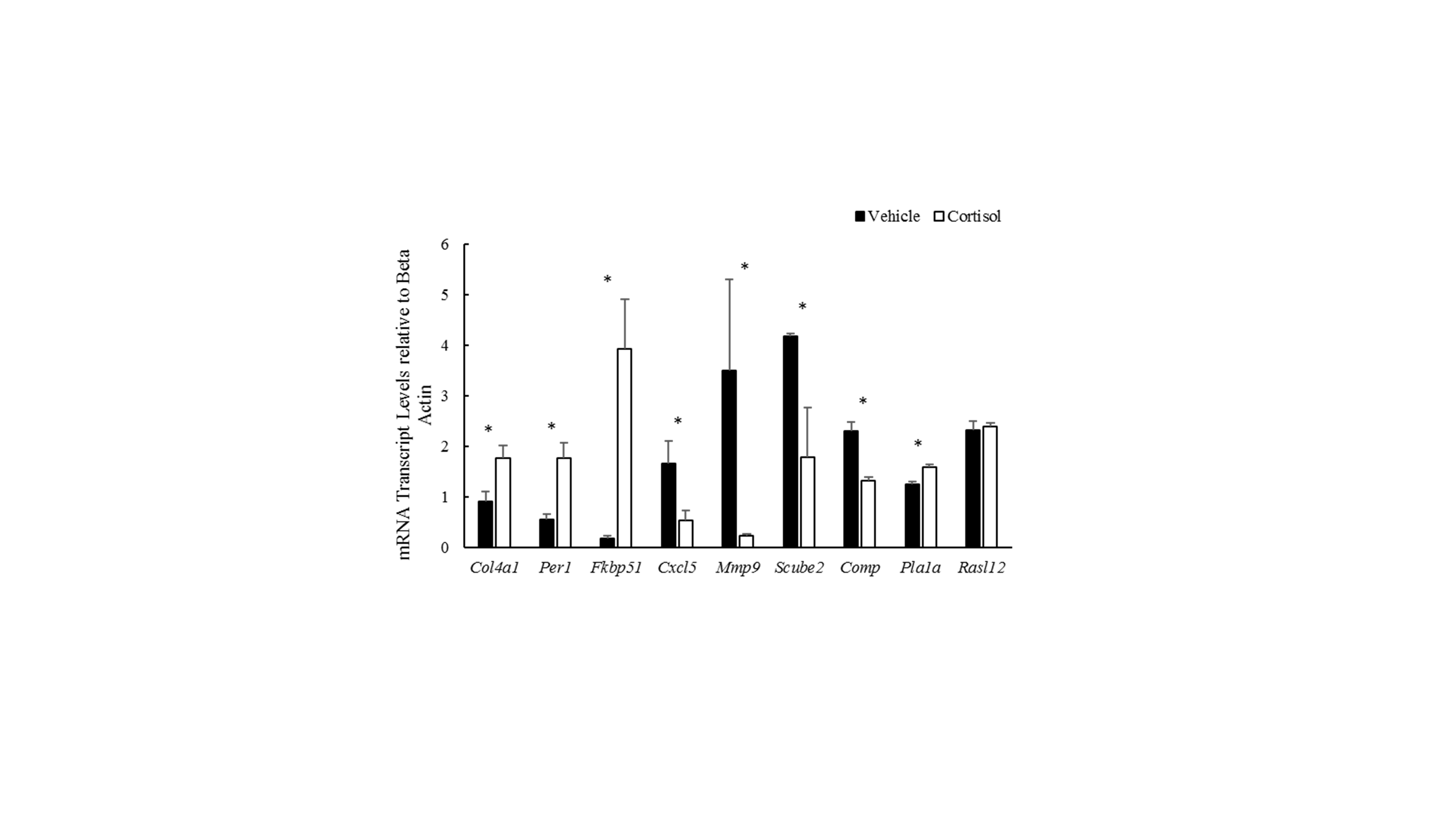

Supplement: S2 Fig — Next generation sequencing analysis was validated by quantification of three genes known to be up-regulated by cortisol and found to be up-regulated in our sequencing analysis (Collagen, type XIV, alpha 1 (Col4a), Period 1 (Per1) and FK506 binding protein 5 (Fkbp5)) and two genes that are known to be down-regulated by cortisol and were down-regulated in this our sequencing analysis (Matrix metalloprotease 9 (Mmp9)) and chemokine (C-X-C motif) ligand 5 (Cxcl5)) in the murine model. In addition we validated genes that were differentially expressed in the mouse compared to the horse in our sequencing analysis (Scube2 (Signal Peptide, CUB Domain, EGF-Like 2) Comp (cartilage oligomeric matrix protein), Pla1a (Phospholipase A1 member A) and Rasl12 (RAS-like, family 12). Data are mean ± SEM for and were analysed by Student’s t-test. * = P<0.05. (TIF) [file pone.0192746.s002.tif]
