# Supplementary material for: Species-specific regulation of angiogenesis by glucocorticoids reveals contrasting effects on inflammatory and angiogenic pathways
Source: PLoS One. 2018 Feb 15;13(2):e0192746. doi: 10.1371/journal.pone.0192746 (PMC5813970; doi:10.1371/journal.pone.0192746)
Supplement: S1 File — Table A. Clinical and biochemical data from healthy horses and those with laminitis. (Vessels were cultured in Matrigel for quantification of angiogenic response). Table B. Clinical and biochemical data from healthy horses and those with laminitis. (Vessels were cultured in collagen for next generation sequencing). Table C. Murine primer sequences for PCRS4. Table D. New vessel outgrowths from laminar vessels of healthy horses and those with laminitis cultured in Matrigel or Type 1 Collagen at day 3. Table E. New vessel outgrowths from laminar vessels of healthy horses and those with laminitis cultured in Matrigel or Type 1 Collagen at day 7. Table F. New vessel outgrowths from laminar arteries and laminar veins of healthy horses and those with laminitis cultured in Matrigel at day 3. Table G. New vessel outgrowths from laminar arteries and laminar veins of healthy horses and those with laminitis cultured in Matrigel at day 7. (DOCX) [file pone.0192746.s003.docx]

**S1 File**

**Table A Clinical and biochemical data from healthy horses and those with laminitis. (Vessels were cultured in Matrigel for quantification of angiogenic response).**

|  | **Healthy (Matrigel)**  **n = 10** | **Laminitis (Matrigel)**  **n = 6** |
| --- | --- | --- |
| **Age (years)** | 19.1 ± 1.4 | 21.5 ± 2.3 |
| **Insulin (mIU/L)** | 4.1 ± 1.4 | 57.1 ± 29.0* |
| **ACTH (pg/mL)** | 39.6 ± 19.5 | 156.1 ± 23.9* |
| **Cortisol (nmol/L)** | 152.6 ± 12.5 | 161.4 ± 22.8 |

Data are mean ± SEM and the groups were compared using a Student’s t-test or a Mann-Whitney U test (Insulin). * denotes a significant difference between the groups (P>0.05).

**Table B Clinical and biochemical data from healthy horses and those with laminitis (Vessels were cultured in collagen for next generation sequencing).**

|  | **Healthy (Collagen)**  **n=3** | **Laminitis (Collagen)**  **n=3** |
| --- | --- | --- |
| **Age (years)** | 18.9 ± 2.4 | 19.8 ± 2.7 |
| **Insulin (mIU/L)** | 5.8 ± 1.2 | 32.0 ± 12.4 |
| **ACTH (pg/mL)** | 28.2 ± 6.5 | 75.4 ± 19.6 |
| **Cortisol** | 146.2 ± 21.3 | 157.9 ± 16.3 |

Data are mean ± SEM and the groups were compared using a Student’s t-test or a Mann-Whitney U test (Insulin). * denotes a significant difference between the groups (P>0.05).

| **Gene symbol, full name** | **Forward Primer** | **Reverse Primer** | **UPL** |
| --- | --- | --- | --- |
| *Actβ*  (Actin, beta) | accagaggcatacagggaca | ctaaggccaaccgtgaaaag | 64 |
| *Col4a1*  (Collagen, type IV, alpha 1) | agttggaggaatgggcttg | ccagggacaccctgtgag | 80 |
| *Cxcl5*  (C-X-C motif chemokine ligand 5) | cagtgggtttgagaacaccata | ctggaggctcattgtggac | 25 |
| *Fkbp51*  (FK506 binding protein 5) | ccttcttgctccgagcttt | tgttcaagaagttcgcagagc | 69 |
| *Mmp9*  (Matrix metallopeptidase 9) | cagaggtaacccacgtcagc | gggatccaccttctgagactt | 7 |
| *Per1*  (Period circadian clock 1) | acagcagccacggttctc | ggacccaggagtgcacag | 71 |

**Table C Murine primer sequences for PCR**

**Table D New vessel outgrowths from laminar vessels of healthy horses and those with laminitis cultured in Matrigel or Type 1 Collagen at day 3.**

|  | **Healthy:**  **Matrigel (n=10)** | **Healthy:**  **Collagen (n=3)** | **Laminitis:**  **Matrigel (n=6)** | **Laminitis:**  **Collagen (n=3)** |
| --- | --- | --- | --- | --- |
| **DMEM** | 5.5 ± 2.3 | 7.3 ± 3.3 | 9.0 ± 5.4 | 2.3 ± 6.7 |
| **FBS** | 11.5 ± 4.3 | 10.0 ± 5.2 | 13.5 ± 6.1 | 17.5 ± 4.9 |
| **Cortisol** | 56.3 ± 15.0 | 25.0 ± 9.5 | 43.0 ± 21.7 | 44.5 ± 17.3 |
| **Cortisol + FBS** | 108.0 ± 54.5 | 44.0 ± 21.3 | 36.6 ± 17.0 | 40.0 ± 10.6 |

Data are mean number of outgrowths ± SEM. Data were analysed by two way ANOVA and Bonferroni post-hoc test. There was no effect of growth matrix or disease group on response to treatment. n = number of horses.

**Table E New vessel outgrowths from laminar vessels of healthy horses and those with laminitis cultured in Matrigel or Type 1 Collagen at day 7.**

|  | **Healthy:**  **Matrigel (n=10)** | **Healthy:**  **Collagen (n=3)** | **Laminitis:**  **Matrigel (n=6)** | **Laminitis:**  **Collagen (n=3)** |
| --- | --- | --- | --- | --- |
| **DMEM** | 17.0 ± 4.9 | 16.2 ± 8.5 | 14.9 ± 6.5 | 13.0 ± 7.3 |
| **FBS** | 28.2 ± 9.2 | 27.5 ± 4.6 | 27.3 ± 6.9 | 30.8 ± 14.3 |
| **Cortisol** | 188.5 ± 43.6 | 82.5 ± 22.3 | 105.6 ± 38.9 | 73.3 ± 34.7 |
| **Cortisol + FBS** | 249.5 ± 78.5 | 172.5 ± 32.7 | 117.5 ± 45.3 | 130.0 ± 34.8 |

Data are mean number of outgrowths ± SEM. Data were analysed by two way ANOVA and Bonferroni post-hoc test. There was no effect of growth matrix or disease group on response to treatment. n = number of horses.

**Table F New vessel outgrowths from laminar arteries and laminar veins of healthy horses and those with laminitis cultured in Matrigel at day 3.**

|  | **Healthy:**  **Artery (n=4)** | **Healthy:**  **Vein (n=6)** | **Laminitis:**  **Artery (n=3)** | **Laminitis:**  **Vein (n=3)** |
| --- | --- | --- | --- | --- |
| **DMEM** | 21.0 ± 4.9 | 16.2 ± 8.0 | 12.4 ± 3.4 | 19.9 ± 6.3 |
| **FBS** | 18.2 ± 8.7 | 26.3 ± 7.5 | 21.5 ± 8.5 | 30.0 ± 10.6 |
| **Cortisol** | 48.3 ± 15.9 | 65.2 ± 30.4 | 58.4 ± 19.4 | 61.7 ± 17.9 |
| **Cortisol + FBS** | 99.4 ± 34.5 | 82.5 ± 29.3 | 36.6 ± 18.5 | 72.0 ± 11.6 |

Data are mean number of outgrowths ± SEM. Data were analysed by two-way ANOVA and Bonferroni post-hoc test. There was no effect of vessel type or disease group on response to treatment. (n= number of horses).

**Table G New vessel outgrowths from laminar arteries and laminar veins of healthy horses and those with laminitis cultured in Matrigel at day 7.**

|  | **Healthy:**  **Artery (n=4)** | **Healthy:**  **Vein (n=6)** | **Laminitis:**  **Artery (n=3)** | **Laminitis:**  **Vein (n=3)** |
| --- | --- | --- | --- | --- |
| **DMEM** | 15.0 ± 7.9 | 18.2 ± 7.5 | 15.4 ± 9.2 | 13.0 ± 7.3 |
| **FBS** | 37.2 ± 14.2 | 24.5 ± 7.6 | 18.5 ± 3.5 | 33.0 ± 12.6 |
| **Cortisol** | 167.5 ± 35.6 | 142.5 ± 52.3 | 110.6 ± 33.9 | 93.3 ± 24.1 |
| **Cortisol + FBS** | 221.5 ± 86.5 | 182.5 ± 72.4 | 207.5 ± 43.6 | 183.0 ± 44.3 |

Data are mean number of outgrowths ± SEM. Data were analysed by two way ANOVA and Bonferroni post-hoc test. There was no effect of vessel type or disease group on response to treatment. n = number of horses. (n= number of horses).
